# Supplementary material for: Accurate single-molecule spot detection for image-based spatial transcriptomics with weakly supervised deep learning
Source: bioRxiv. 2024 Feb 5:2023.09.03.556122. Preprint. [Version 3] doi: 10.1101/2023.09.03.556122 (PMC10508757; doi:10.1101/2023.09.03.556122)
Supplement: Supplement 1 [file NIHPP2023.09.03.556122v3-supplement-1.pdf]

bioRxiv preprint doi: https://doi.org/10.1101/2023.09.03.556122; this version posted February 5, 2024. The copyright holder for this preprint (which was not certified by peer review) is the author/funder, who has granted bioRxiv a license to display the preprint in perpetuity. It is made available under aCC-BY 4.0 International license.

6 Supplementary Information

6.1 Supplementary Note 1: Evaluation of generative model performance for the creation of training data

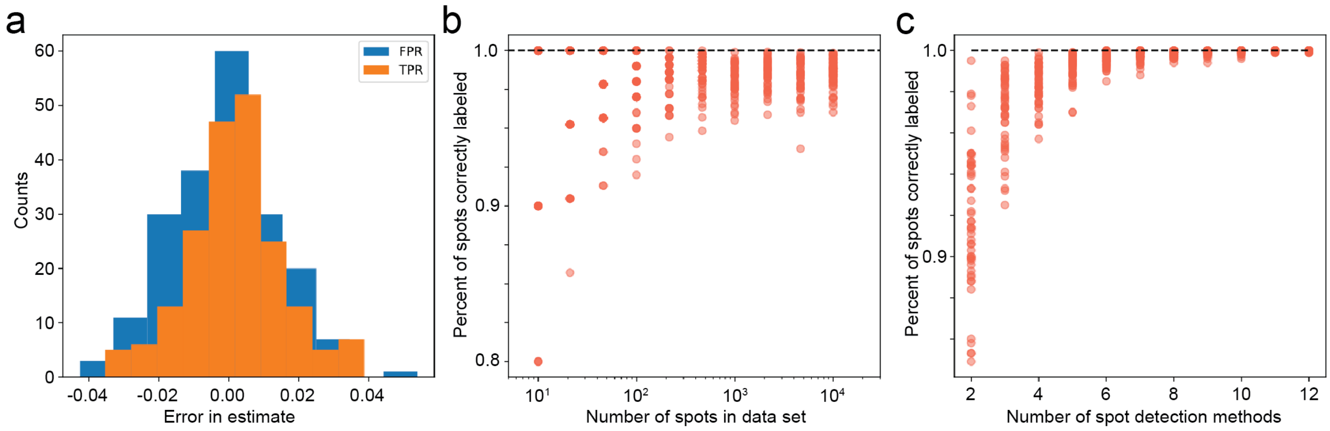

Figure S1: **Benchmarking consensus annotation output of the generative model.** (a) Error distribution for EM estimates of TPR and FPR values for 100 trials with three simulated classical methods. (b) Fraction of simulated detections correctly classified with increasing dataset size (number of spots in the dataset). (c) Fraction of simulated detections correctly classified as a true or false detection by EM for an increasing number of classical spot detection methods used in the EM method.

bioRxiv preprint doi: https://doi.org/10.1101/2023.09.03.556122; this version posted February 5, 2024. The copyright holder for this preprint (which was not certified by peer review) is the author/funder, who has granted bioRxiv a license to display the preprint in perpetuity. It is made available under aCC-BY 4.0 International license.

6.2 Supplementary Note 2: Custom loss function for Polaris' deep learning model for spot detection

We trained the network using a custom loss function composed of a classification loss and a regression loss, which considers the outputs of both of the model's prediction heads. The loss function has the following form:

$$L(y, \hat{y}) = L_{\text{cla}}(C, \hat{C}) + L_{\text{reg}}(R, \hat{R}) \tag{12}$$

where C is the classification head output, R is the regression head output, and $y = (C, R)$. The classification loss is the weighted cross-entropy with inverse class frequency-based weights. The regression loss is given by:

$$L_{\text{reg}}(R, \hat{R}) = \frac{1}{|G_d|} \sum_{i \in G_d} \ell((dy_i, dx_i), (\hat{dy}_i, \hat{dx}_i)) \tag{13}$$

where $R_i = (dy_i, dx_i)$ (i denotes a single pixel), $G_d = \{\text{pixels } i = (i_y, i_x) |$ a spot-containing pixel j exists with $L_\infty(i, j) = \max_{k \in x,y} |i_k - j_k| \leq d\}$, and ℓ is the smooth L_1 function. dx_i is the x-coordinate of the position difference between the nearest spot to pixel i, and pixel i's center. Similarly, dy_i is the y-coordinate of this position difference. d is a configurable parameter that determines the threshold distance from the nearest spot under which the estimated nearest spot's position for that pixel is taken into account in the loss function.

bioRxiv preprint doi: https://doi.org/10.1101/2023.09.03.556122; this version posted February 5, 2024. The copyright holder for this preprint (which was not certified by peer review) is the author/funder, who has granted bioRxiv a license to display the preprint in perpetuity. It is made available under aCC-BY 4.0 International license.

6.3 Supplementary Note 3: Generalization of Polaris' spot detection model to a variety of spot images

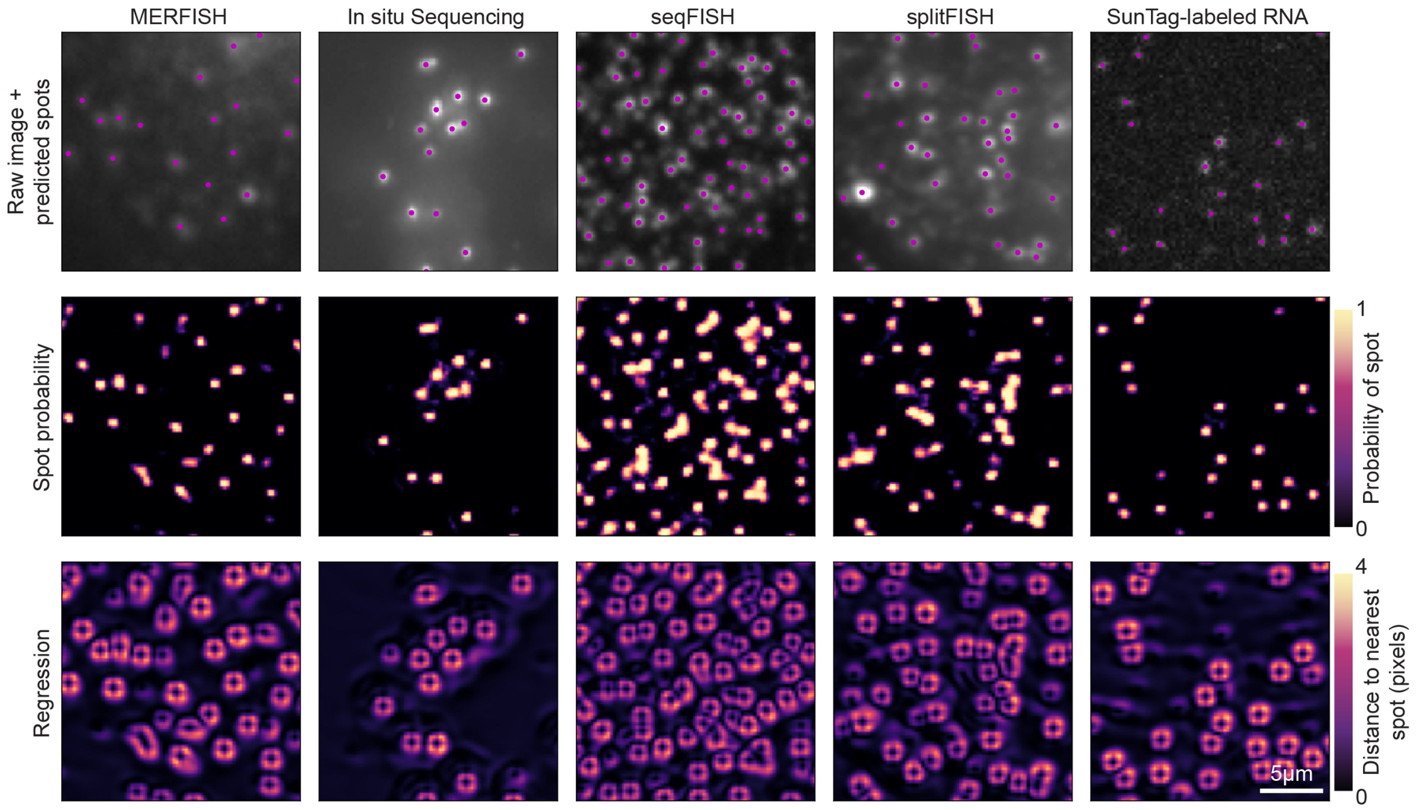

Figure S2: **Polaris' spot detection model generalizes to spot images generated with a variety of single-molecule assays.** The spot probability prediction images encode the pixel-wise spot probability. The regression image is the sum of the square of the subpixel distances to the nearest spot in the x- and y-dimensions. Pixels beyond a threshold value are set to zero. These outputs are used together to generate a set of predicted spot locations with subpixel resolution, plotted over the raw image.

bioRxiv preprint doi: https://doi.org/10.1101/2023.09.03.556122; this version posted February 5, 2024. The copyright holder for this preprint (which was not certified by peer review) is the author/funder, who has granted bioRxiv a license to display the preprint in perpetuity. It is made available under aCC-BY 4.0 International license.

6.4 Supplementary Note 4: Mutual nearest-neighbor method for matching sets of spots

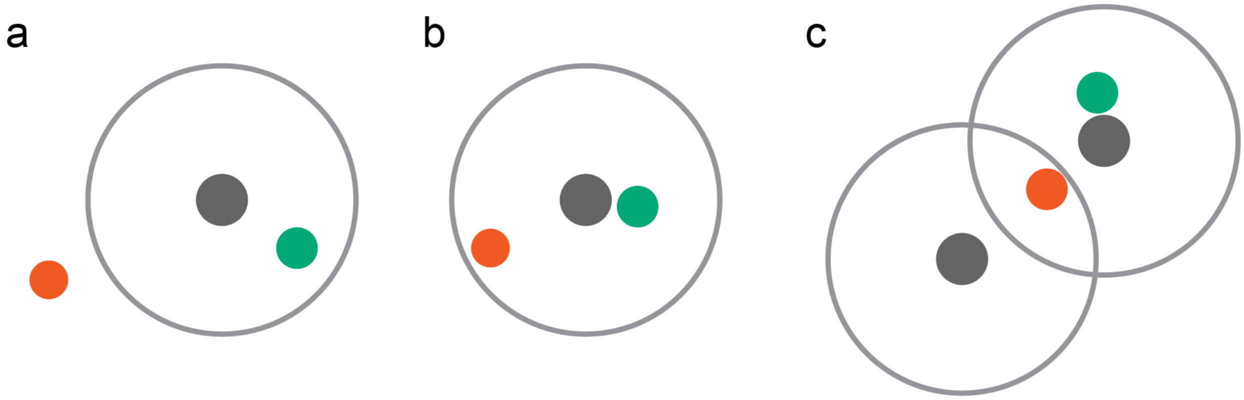

Figure S3: **Example cases handled by a mutual nearest-neighbor matching algorithm.** (a) Example with spots inside and outside the threshold distance to a ground-truth spot. Ground truth spots and their threshold distance are shown in grey. True positive detections are shown in green and false positive detections are shown in orange. (b) Example with two spots inside the threshold distance to a ground-truth spot. (c) Example with two spots within the threshold distance of two ground-truth spots.

bioRxiv preprint doi: https://doi.org/10.1101/2023.09.03.556122; this version posted February 5, 2024. The copyright holder for this preprint (which was not certified by peer review) is the author/funder, who has granted bioRxiv a license to display the preprint in perpetuity. It is made available under aCC-BY 4.0 International license.

6.5 Supplementary Note 5: Inter-algorithm agreement of spot detection results

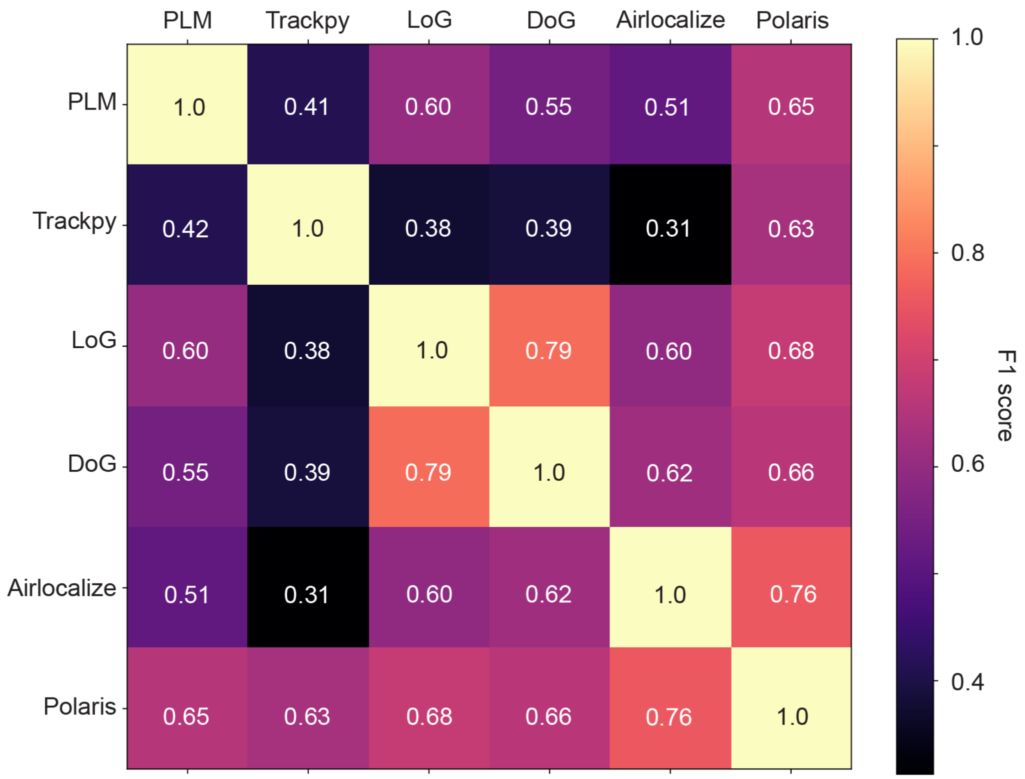

Figure S4: **Quantification of agreement between Polaris' deep learning model and different classical spot detection methods.** The benchmarked methods include maximum-intensity filtering (PLM), the Crocker-Grier centroid-finding algorithm (Trackpy), Laplacian of Gaussian (LoG), difference of Gaussians (DoG), Airlocalize, and Polaris.

bioRxiv preprint doi: https://doi.org/10.1101/2023.09.03.556122; this version posted February 5, 2024. The copyright holder for this preprint (which was not certified by peer review) is the author/funder, who has granted bioRxiv a license to display the preprint in perpetuity. It is made available under aCC-BY 4.0 International license.

6.6 Supplementary Note 6: Benchmarking the receptive field of Polaris' spot detection model

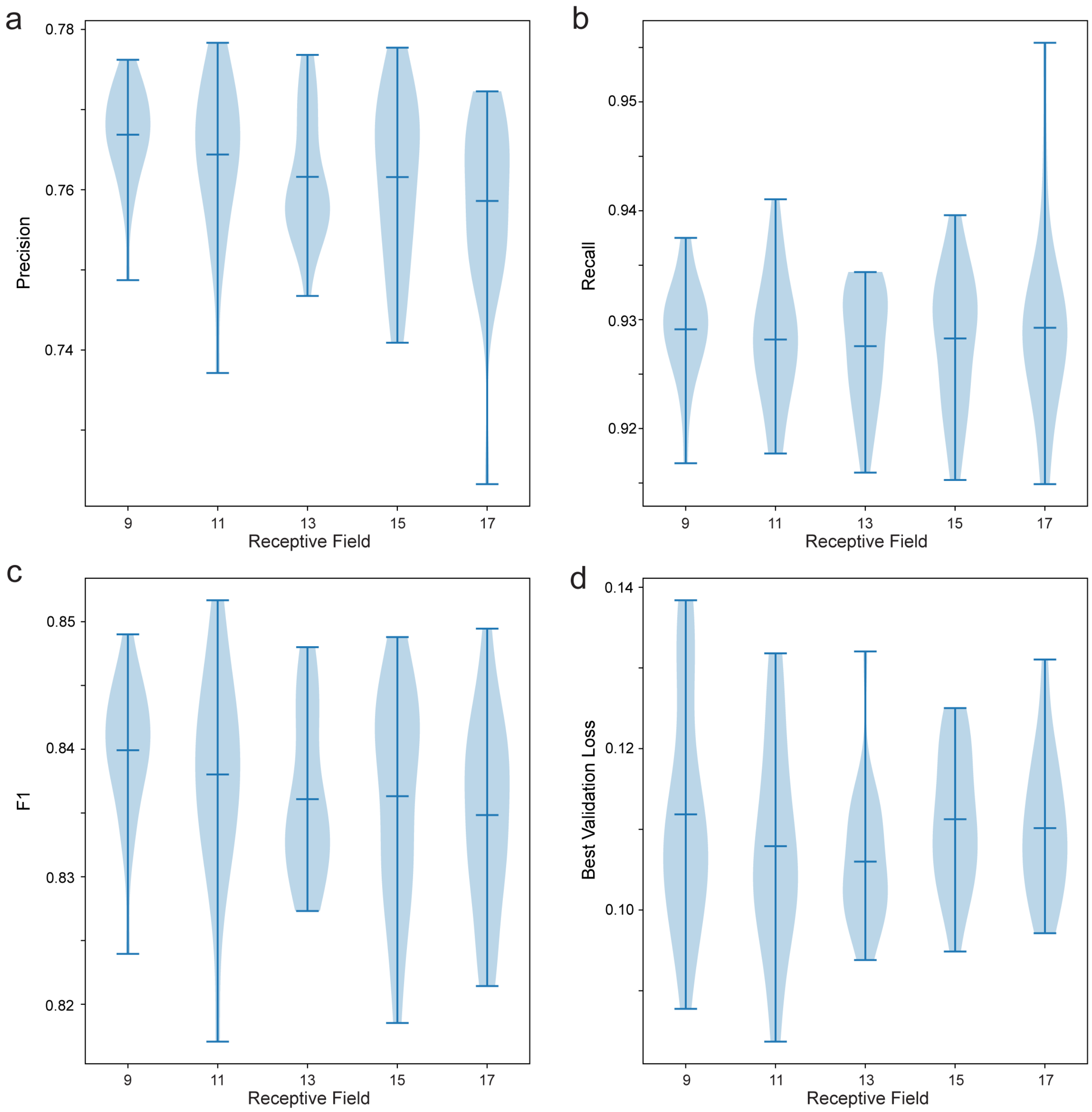

Figure S5: **Benchmarking the receptive field parameter of Polaris' spot detection model.** (a-d) Violin plot quantifying the performance metrics ((a) precision, (b) recall, (c) F1, (d) best validation loss during training) for models trained with different values for receptive field of Polaris' spot detection model. n=24 trained models per receptive field condition.

bioRxiv preprint doi: https://doi.org/10.1101/2023.09.03.556122; this version posted February 5, 2024. The copyright holder for this preprint (which was not certified by peer review) is the author/funder, who has granted bioRxiv a license to display the preprint in perpetuity. It is made available under aCC-BY 4.0 International license.

6.7 Supplementary Note 7: Benchmarking Polaris' spot detection model on simulated images with ranging spot characteristics

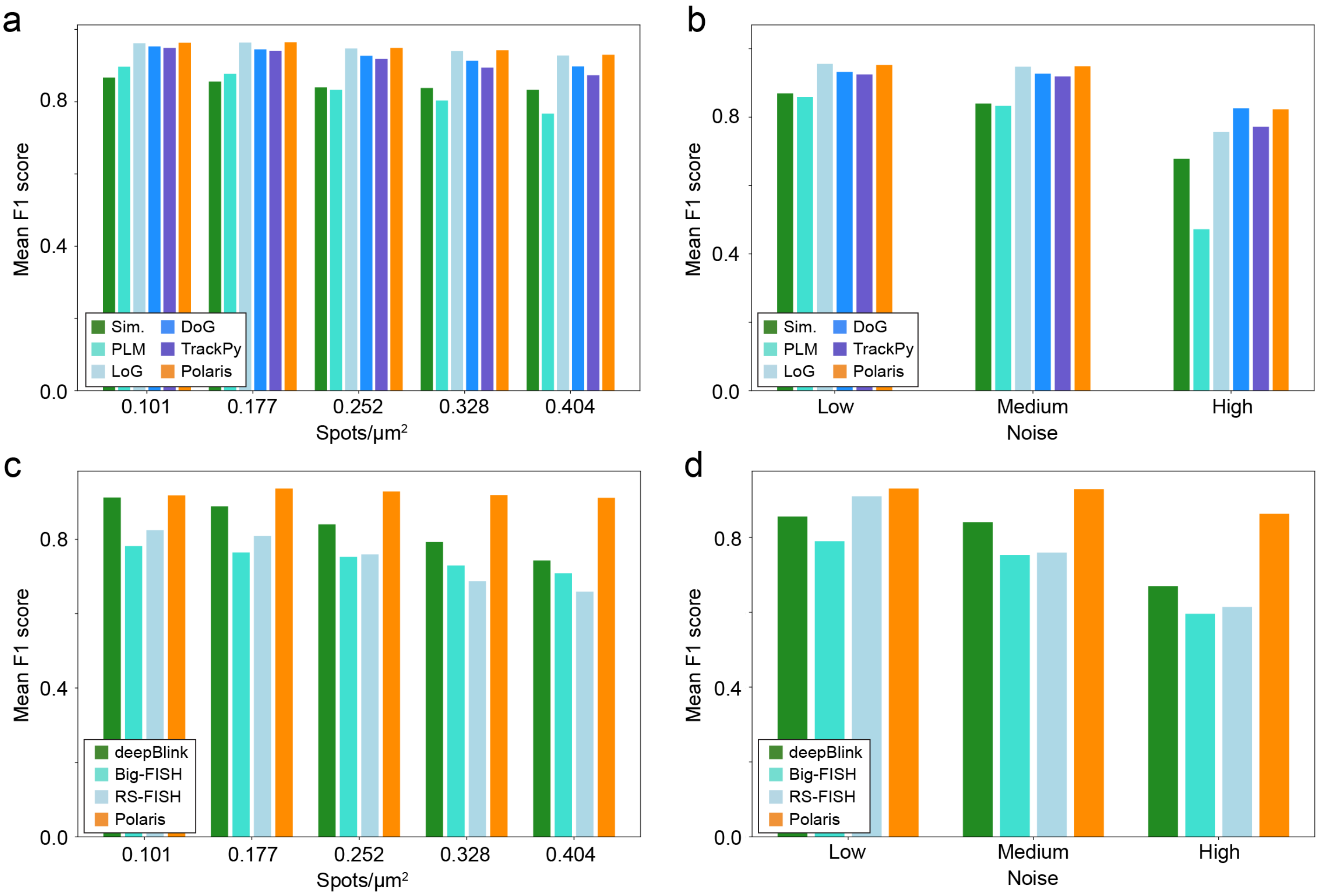

Figure S6: **Benchmarking model performance on simulated spot images with a range of spot intensities and densities.** (a) Performance quantification for models with Polaris' deep learning architecture trained with various datasets predicting on images with a range of spot density. (b) Performance quantification for models with Polaris' deep learning architecture trained with various datasets predicting on images with a range of levels of simulated noise. The low noise condition corresponds to a signal-to-noise ratio of greater than approximately 16. The medium noise condition corresponds to a SNR of approximately 8-15. The high noise condition corresponds to a SNR of approximately 3-7. (c) Performance quantification for various spot detection methods on images with a range of spot densities. (d) Performance quantification for various spot detection methods on images with a range of levels of simulated noise. The SNR ranges for each noise condition are the same as those in (b).

bioRxiv preprint doi: https://doi.org/10.1101/2023.09.03.556122; this version posted February 5, 2024. The copyright holder for this preprint (which was not certified by peer review) is the author/funder, who has granted bioRxiv a license to display the preprint in perpetuity. It is made available under aCC-BY 4.0 International license.

6.8 Supplementary Note 8: Robustness of Polaris' barcode decoding method to dropout

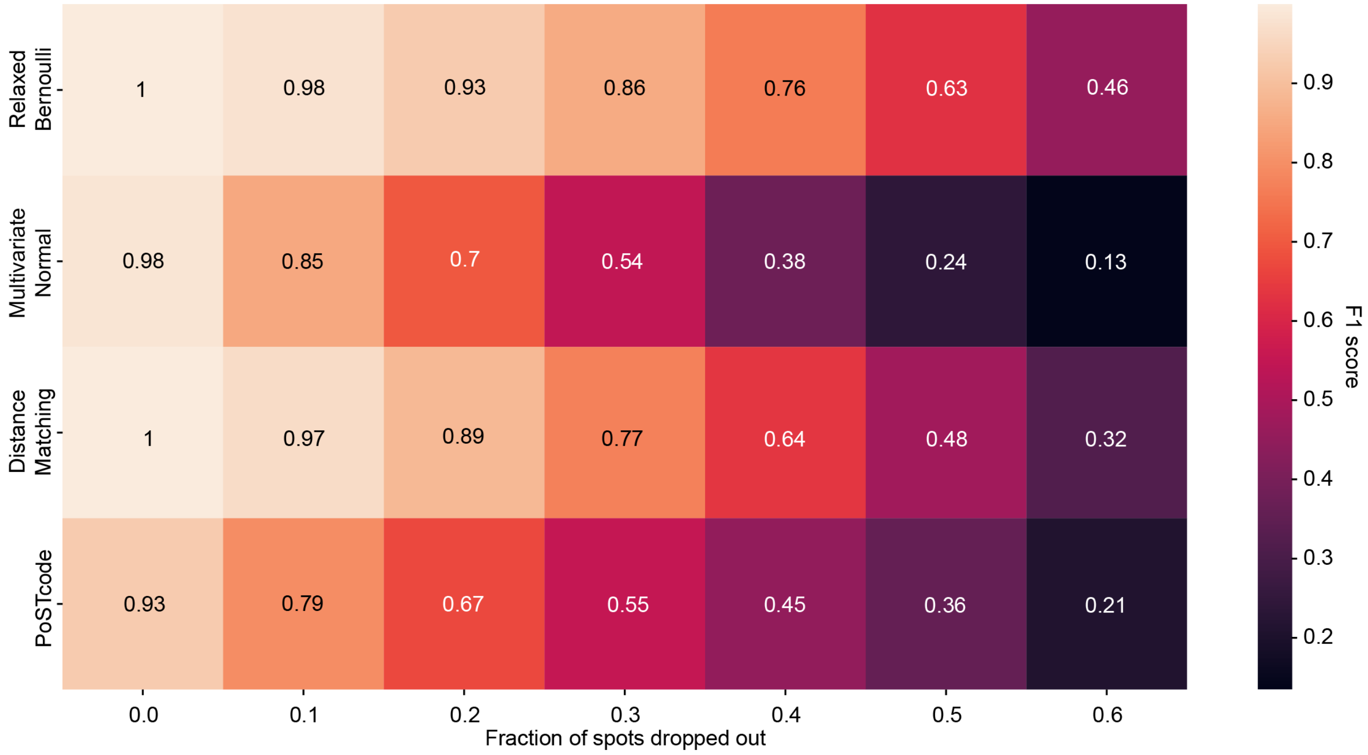

Figure S7: **Benchmarking of the robustness of gene decoding methods to dropout.** Quantification of F1 score for four barcode decoding methods (a graphical model of relaxed Bernoulli distributions, a graphical model of multivariate normal distributions, Hamming distance matching, and PoSTcode) for simulated barcode pixel values with a range of dropout rates.

bioRxiv preprint doi: https://doi.org/10.1101/2023.09.03.556122; this version posted February 5, 2024. The copyright holder for this preprint (which was not certified by peer review) is the author/funder, who has granted bioRxiv a license to display the preprint in perpetuity. It is made available under aCC-BY 4.0 International license.

6.9 Supplementary Note 9: Benchmarking Polaris' performance on various multiplexed FISH image sets

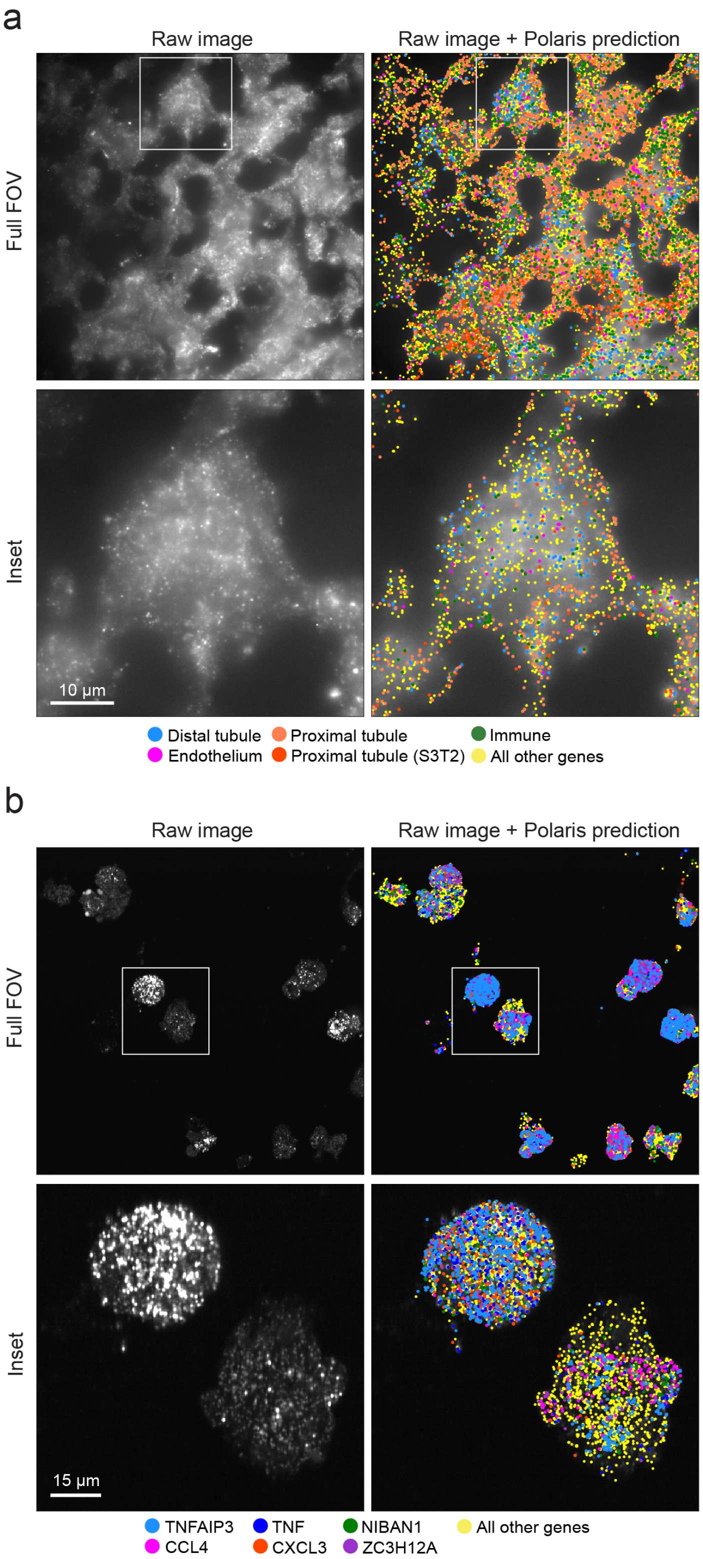

Figure S8: **Demonstration of Polaris' performance on a MERFISH and seqFISH data.** (a) Example Polaris prediction for a MERFISH experiment in a mouse kidney tissue sample (Liu, et al. 2022). (b) Example Polaris prediction for a seqFISH experiment in a macrophage cell culture sample. The spot colors of the Polaris prediction in (a,b) denote the predicted gene identities. The inset image location is defined by the white box in the full field of view (FOV).

bioRxiv preprint doi: https://doi.org/10.1101/2023.09.03.556122; this version posted February 5, 2024. The copyright holder for this preprint (which was not certified by peer review) is the author/funder, who has granted bioRxiv a license to display the preprint in perpetuity. It is made available under aCC-BY 4.0 International license.

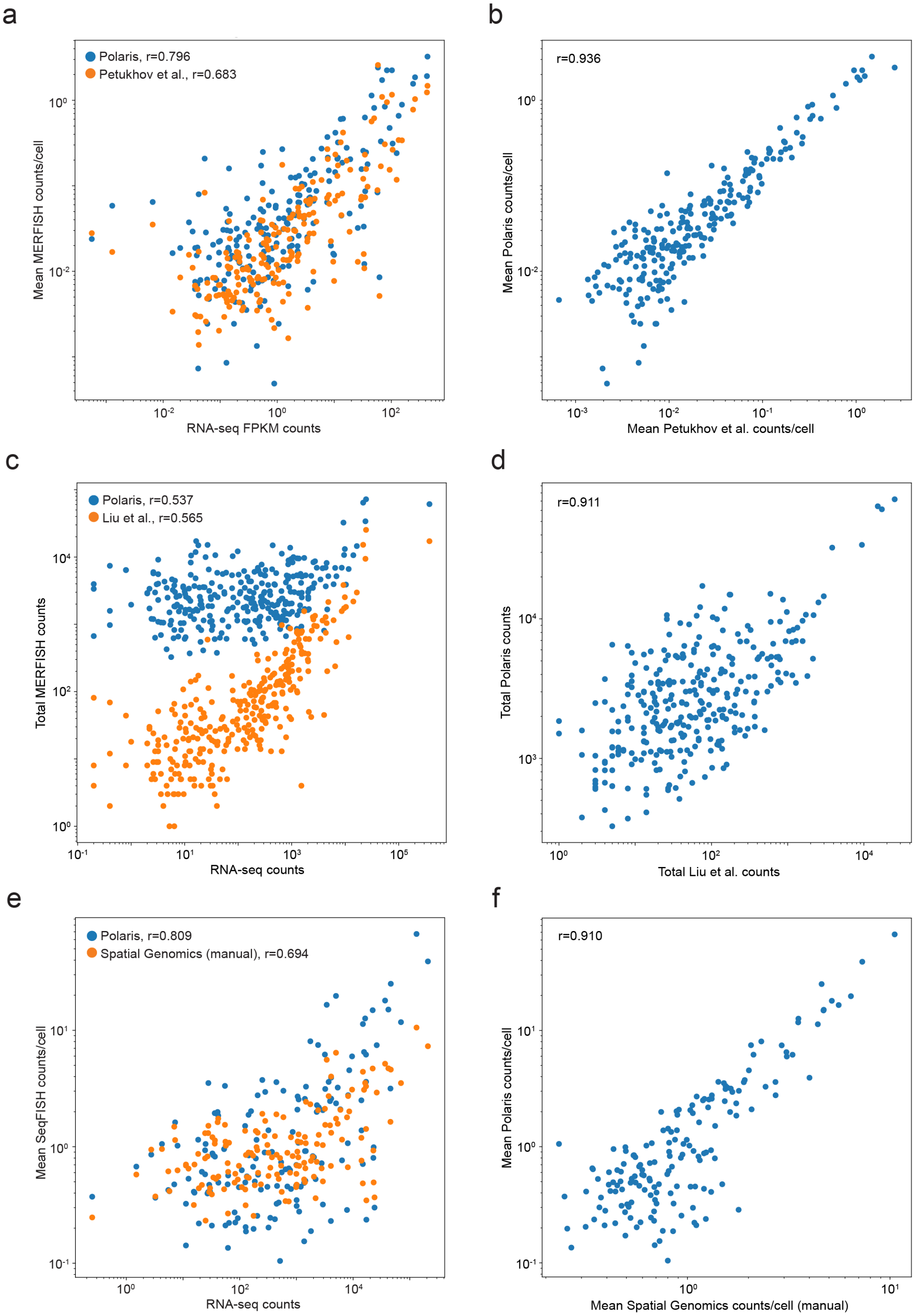

Figure S9: **Correlation of Polaris' quantification of MERFISH data with other quantification methods.** (a,c) Scatter plot plotted in logspace, comparing gene expression counts quantified with MERFISH with counts measured with RNA-seq. (b,d) Scatter plot plotted in logspace, comparing previously published MERFISH gene expression counts with counts quantified with Polaris. (e) Scatter plot plotted in logspace, comparing mean gene expression counts per cell quantified with seqFISH with counts measured with RNA-seq. (f) Scatter plot plotted in logspace, comparing mean gene counts per cell obtained by manual analysis of seqFISH data with gene counts quantified with Polaris.

bioRxiv preprint doi: https://doi.org/10.1101/2023.09.03.556122; this version posted February 5, 2024. The copyright holder for this preprint (which was not certified by peer review) is the author/funder, who has granted bioRxiv a license to display the preprint in perpetuity. It is made available under aCC-BY 4.0 International license.

6.10 Supplementary Note 10: Demonstration of Polaris' performance decoding an ISS barcode library

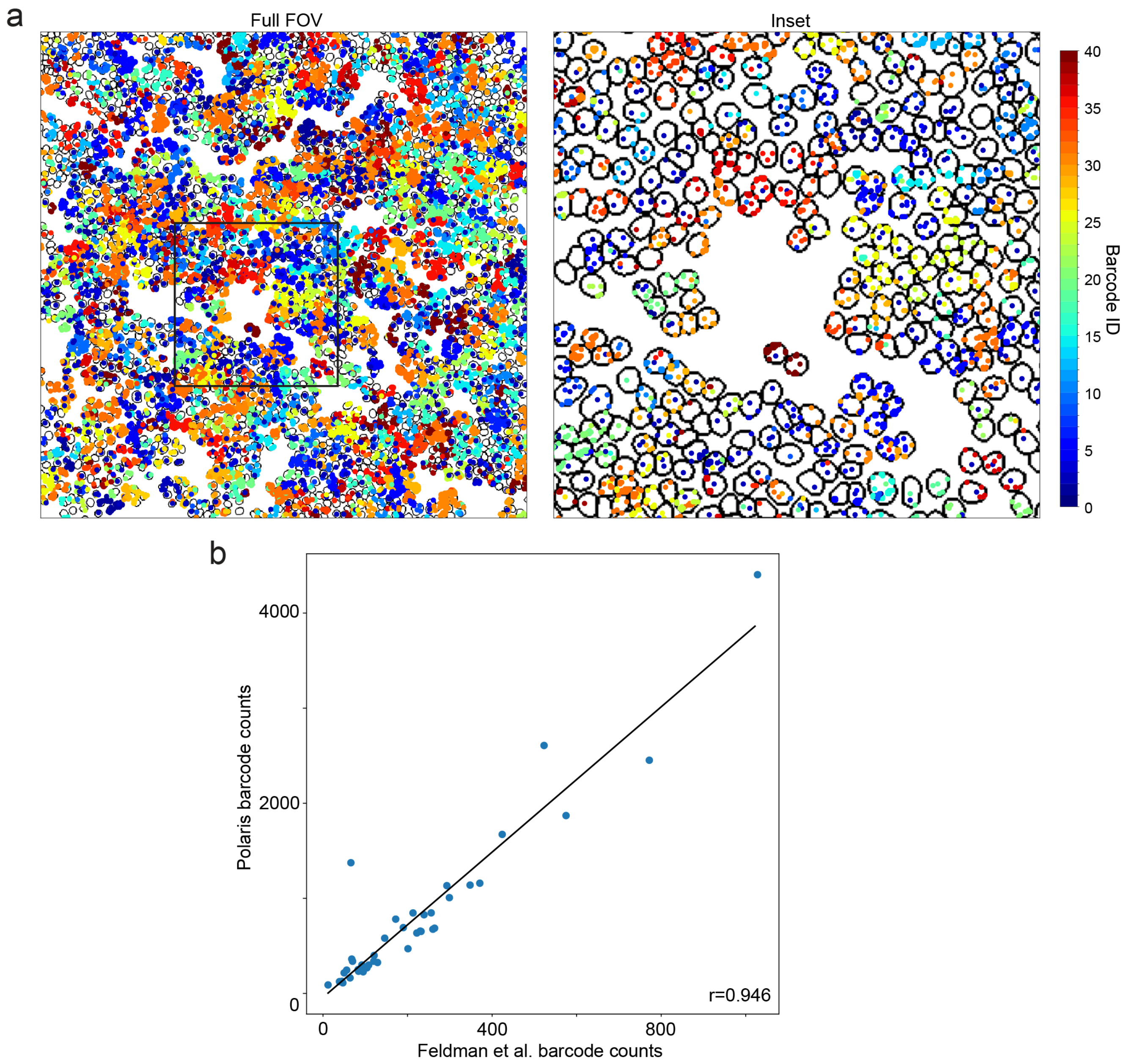

Figure S10: **Demonstration of Polaris' performance on an ISS dataset in HeLa cells.** (a) Example Polaris prediction for the ISS sample. The spot colors correspond with barcode identities. The inset location is defined by the black box in the full field of view (FOV). (b) Scatter plot correlating total counts for each barcode decoded by the original published analysis with counts quantified by Polaris (r=0.946).
