# Supplementary material for: Accurate single-molecule spot detection for image-based spatial transcriptomics with weakly supervised deep learning
Source: bioRxiv. 2023 Sep 8:2023.09.03.556122. Preprint. [Version 2] doi: 10.1101/2023.09.03.556122 (PMC10508757; doi:10.1101/2023.09.03.556122)
Supplement: Supplementary file 1 [file NIHPP2023.09.03.556122v2-supplement-1.pdf]

bioRxiv preprint doi: https://doi.org/10.1101/2023.09.03.556122; this version posted September 8, 2023. The copyright holder for this preprint (which was not certified by peer review) is the author/funder, who has granted bioRxiv a license to display the preprint in perpetuity. It is made available under aCC-BY 4.0 International license.

5 Supplementary Information

5.1 Supplementary Note 1: Construction of training data for Polaris' spot detection model

5.1.1 Overview

To construct a training dataset for Polaris' spot detection model, we assembled a set of representative images from sequential fluorescence in situ hybridization (seqFISH)[10], multiplexed error-robust FISH (MERFISH)[29,35], and SunTag labeled imaging data[36]. We detected the location of these spots using the four classical spot detection algorithms described in the methods section. Here, we refer to each of these classical spot detection algorithms as "annotators". The parameters of these methods were manually fine-tuned on a per-image basis to optimize the spot detection results for each image. To generate subpixel resolution, we performed Gaussian fitting on the pixel values surrounding the spot location to estimate its subpixel location, as described in the methods section.

To generate consensus spot annotations, we fit a generative model to the detections generated by the four annotators (Supplementary Fig. S1). To determine which annotators detected each spot in an image, the detections from all annotators are first grouped by proximity into clusters. We presume that the detections closer than a defined threshold of 1.5 pixels have derived from the same spot in the image. Each cluster is allowed to contain only one detection from each annotator. If a cluster contains more than one detection from an annotator, the centroid of the cluster is determined, and the detection from that annotator closest to the centroid is included in the cluster. All other detections from that annotator are then separated into new clusters.

The generative model characterizes annotators with two parameters:

1. True positive rate (TPR), which is an annotator's probability of detecting a ground-truth true spot
2. False positive rate (FPR), which is an annotator's probability of detecting a ground-truth false spot

The generative model characterizes detected spots by the probability of corresponding to a "true" spot (p(TP)). Said another way, p(TP) is the probability that a spot is a true spot given that it is detected. The probability that a detection corresponds to a "false" spot (p(FP)) has a value of $1-p$(TP). The generative model is provided an initial guess for the TPR and FPR of each classical algorithm and a matrix, which we name the "detection information matrix". This matrix of annotation data, $x = x_{ic i=1,\dots,n}$ consists of binary variables, x_{ic}, which are equal to 1 if annotator i detected cluster c, and 0 if not. We define z_c to be a binary variable indicating if cluster c is a true spot or not (1 if true, 0 if not). We assume that each annotator i produces Bernoulli distributed annotations. Therefore, for every cluster c and annotator i, the distribution of x_{ic} given the cluster assignment z_c and annotator characteristics $\theta_i(z_c)$ is a Bernoulli distribution:

$$P(x_{ic} = 1|z_c, \theta_i(z_c) = \theta_i(z_c)^{x_{ic}}(1 - \theta_i(z_c))^{1-x_{ic}} \tag{1}$$

We assume the variables x_{ic} are independent to obtain that the probability to observe the data $x = x_{ic}$, given θ_i and z_c is

$$P(\{x_{ic}\}|\{z_c\}, \{\theta_i\}) = \prod_i \prod_c \theta_i(z_c)^{x_{ic}}(1 - \theta_i(z_c))^{1-x_{ic}} \tag{2}$$

bioRxiv preprint doi: https://doi.org/10.1101/2023.09.03.556122; this version posted September 8, 2023. The copyright holder for this preprint (which was not certified by peer review) is the author/funder, who has granted bioRxiv a license to display the preprint in perpetuity. It is made available under aCC-BY 4.0 International license.

We fit our generative model to our spot detection data using the expectation-maximization (EM) algorithm[38]. The EM algorithm iteratively performs three steps. First, the $p(\text{TP})$ and $p(\text{FP})$ values of each detection cluster are estimated given the TPR and FPR for each spot detection algorithm. Second, these probabilities are used to calculate the expectation value for the number of true positives ($\mathbb{E}(\text{TP})$), false positives ($\mathbb{E}(\text{FP})$), true negatives ($\mathbb{E}(\text{TN})$), and false negatives ($\mathbb{E}(\text{FN})$) from each algorithm. These two steps together comprise the expectation step. Third, these expectation values are used to update the estimated TPR and FPR for each algorithm. This step comprises the maximization step. These steps are performed iteratively until the estimate for the $p(\text{TP})$ value of the cluster converges. If the resulting $p(\text{TP})$ of a cluster exceeds a probability threshold of 0.9, the cluster is included in the final consensus set of spot annotations. The location of the consensus spot is taken to be at the centroid of the cluster, because each annotator is assumed to have some random localization error.

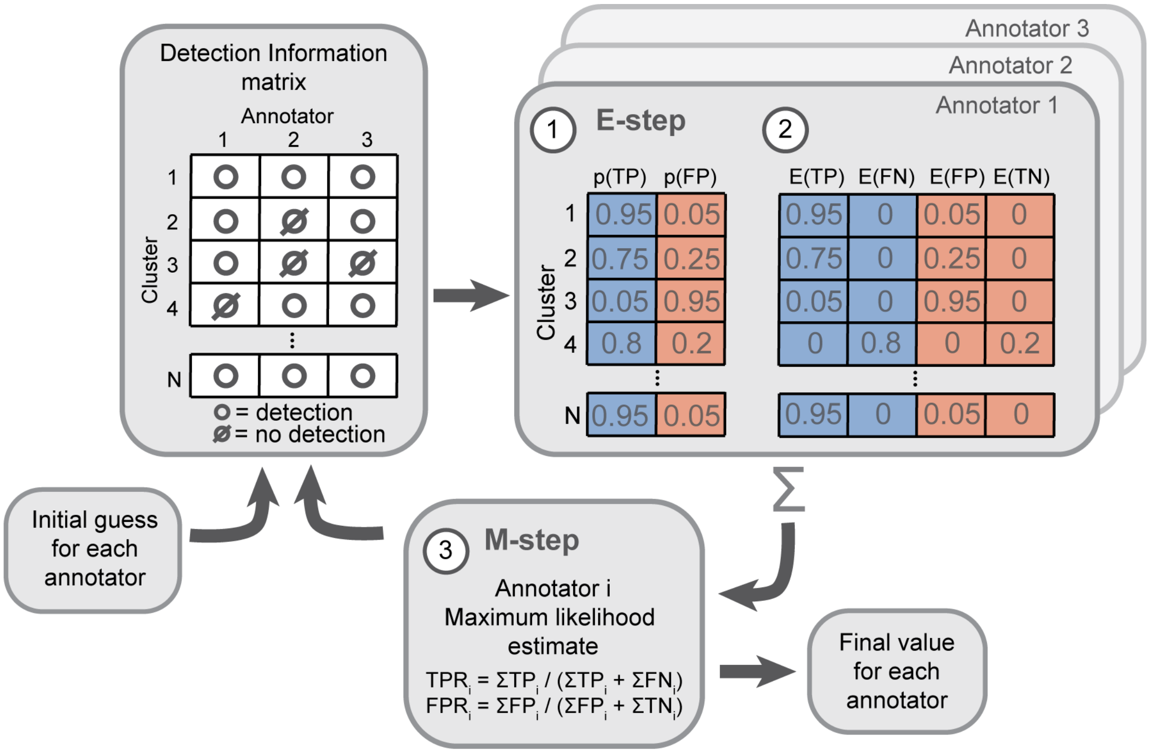

Figure S1: **Schematic diagram of the EM method for consensus spot annotation creation.** The method consists of three steps: (1) Bayesian estimation of $p(\text{TP})$ and $p(\text{FP})$. (2) Calculation of expectation values for the number of true positives (TPs), false negatives (FNs), false positives (FPs), and true negatives (TNs). (3) Update of the maximum likelihood estimate for the TPR and FPR values of each spot detection method.

5.1.2 Computation for the expectation step

We define the probability of a detection being a true or false detection with Bayes' theorem:

$$p(Z|\text{data}, \theta) = \frac{p(\text{data}|Z, \theta)p(Z)}{p(\text{data}|\theta)} \tag{3}$$

where Z is the identity of the spot as a true or false detection, *data* indicates which annotators detected which clusters, and θ is the TPR or FPR of each method. The term $p(Z)$ is the prior probability of a spot being a true or false detection. We

bioRxiv preprint doi: https://doi.org/10.1101/2023.09.03.556122; this version posted September 8, 2023. The copyright holder for this preprint (which was not certified by peer review) is the author/funder, who has granted bioRxiv a license to display the preprint in perpetuity. It is made available under aCC-BY 4.0 International license.

can use the least informative value for the prior by setting $p(Z) = 1/2$, indicating an equal probability that a spot is a true or false detection. The term $p(\text{data}|\theta)$ can be expressed as:

$$p(\text{data}|\theta) = \sum_{Z} p(\text{data}|Z, \theta)p(Z) \tag{4}$$

Therefore, the probability $p(Z|\text{data}, \theta)$ can be written as:

$$\begin{aligned} p(Z|\text{data}, \theta) &= \frac{p(\text{data}|Z, \theta)p(Z)}{p(\text{data}|\theta)} && (5)\\ &= \frac{p(\text{data}|Z, \theta)p(Z)}{\sum_Z p(\text{data}|Z, \theta)p(Z)} && (6)\\ &= \frac{p(\text{data}|Z, \theta) \cdot \frac{1}{2}}{\sum_Z p(\text{data}|Z, \theta) \cdot \frac{1}{2}} && (7)\\ &= \frac{p(\text{data}|Z, \theta)}{\sum_Z p(\text{data}|Z, \theta)} && (8) \end{aligned}$$

This equation states that the likelihood of each possible label (e.g., true or false) is normalized by the total likelihood of both labels to calculate the probability that a cluster is a true or false detection.

We can use Bayes' theorem to calculate the probability of observing *data* given the TPR and FPR of each method. To offer a concrete example, consider the following three situations for a hypothetical set of three annotators. For a "true detection," the probability that all three annotators detect the spot is given by the product of the TPRs of each method:

$$p(\text{data}|Z, \theta) = \prod_{i=1}^{3} \text{TPR}_i \tag{9}$$

Alternatively, the probability that the first two annotators detect a ground-truth true spot while the third annotator (incorrectly) does not is given by the following equation:

$$p(\text{data}|Z, \theta) = \text{TPR}_1 \cdot \text{TPR}_2 \cdot (1 - \text{TPR}_3) \tag{10}$$

Similarly, the probability that the first two annotators incorrectly detect a ground-truth false spot while the last annotator (correctly) does not is given by the following equation:

$$p(\text{data}|Z, \theta) = \text{FPR}_1 \cdot \text{FPR}_2 \cdot (1 - \text{FPR}_3) \tag{11}$$

We can then calculate $\mathbb{E}(\text{TP})$, $\mathbb{E}(\text{FN})$, $\mathbb{E}(\text{FP})$, and $\mathbb{E}(\text{TN})$ for each annotator. Two scenarios can arise in calculating these values.

1. If an annotator detects a spot in a particular cluster, $\mathbb{E}(\text{TP})$ for that method is equal to $p(\text{TP})$ for that cluster, and $\mathbb{E}(\text{FP})$ for that annotator is equal to $p(\text{FP})$ for that cluster. $\mathbb{E}(\text{TN})$ and $\mathbb{E}(\text{FN})$ are set to zero.

2. If an annotator does not detect a spot in a particular cluster, $\mathbb{E}(\text{TN})$ for that annotator is equal to $p(\text{FP})$ for that cluster, and $\mathbb{E}(\text{FN})$ for that annotator is equal to $p(\text{TP})$ for that cluster. $\mathbb{E}(\text{TP})$ and $\mathbb{E}(\text{FP})$ are set to zero.

bioRxiv preprint doi: https://doi.org/10.1101/2023.09.03.556122; this version posted September 8, 2023. The copyright holder for this preprint (which was not certified by peer review) is the author/funder, who has granted bioRxiv a license to display the preprint in perpetuity. It is made available under aCC-BY 4.0 International license.

5.1.3 Computation for the maximization step

We sum $\mathbb{E}(\mathrm{TP})$, $\mathbb{E}(\mathrm{FN})$, $\mathbb{E}(\mathrm{FP})$, and $\mathbb{E}(\mathrm{TN})$ across all clusters to calculate an updated maximum likelihood estimate for TPR_i and FPR_i for method i with equations of the following form:

$$\mathrm{TPR}_i = \frac{\sum_i \mathbb{E}(\mathrm{TP}_i)}{\sum_i \mathbb{E}(\mathrm{TP}_i) + \sum_i \mathbb{E}(\mathrm{FN}_i)} \tag{12}$$

$$\mathrm{FPR}_i = \frac{\sum_i \mathbb{E}(\mathrm{FP}_i)}{\sum_i \mathbb{E}(\mathrm{FP}_i) + \sum_i \mathbb{E}(\mathrm{TN}_i)} \tag{13}$$

5.1.4 Evaluation of generative model performance for the creation of training data

We performed simulated experiments to demonstrate the accuracy of our generative model output by simulating spot detection methods with varying TPR and FPR values and simulating spot identities as true or false spots. These simulated experiments do not involve simulated images or application of classical spot detection methods, but instead involve simulating annotations in the form of a detection information matrix. These experiments demonstrate that the EM algorithm can be applied to accurately find consensus among noisy detections.

We demonstrate that the generative model outputs TPR and FPR estimates for classical methods that are close to their actual values. Using the simulated detection information matrix for simulated spot detection methods with different TPR and FPR values, we determined the error distribution for the TPR and FPR estimates of the simulated spot detection methods based on 200 trials of EM parameter estimation. For the simulated spot detection methods, the TPR and FPR values were sampled from uniform distributions centered at 0.9 and 0.1, respectively (Supplementary Fig. S2a). Using four simulated spot detection methods, we investigated the relationship between the accuracy of the generative model output and the number of spots in the input data set (Supplementary Fig. S2b). The fraction of correctly labeled detections increases with the number of spot detections in the dataset, with the accuracy exceeding 95% over the typical range of dataset sizes for training Polaris' deep learning model. Additionally, using a simulated dataset size of 1000 spots, we found that the accuracy of the generative model output improves with the number of spot detection methods used to create the consensus spot locations, approaching 100% (Supplementary Fig. S2c). In these experiments, we ran 50 independent simulations per condition in which TPRs and FPRs were sampled from uniform distributions with a range of 0.2, centered at 0.9 and 0.1, respectively.

bioRxiv preprint doi: https://doi.org/10.1101/2023.09.03.556122; this version posted September 8, 2023. The copyright holder for this preprint (which was not certified by peer review) is the author/funder, who has granted bioRxiv a license to display the preprint in perpetuity. It is made available under aCC-BY 4.0 International license.

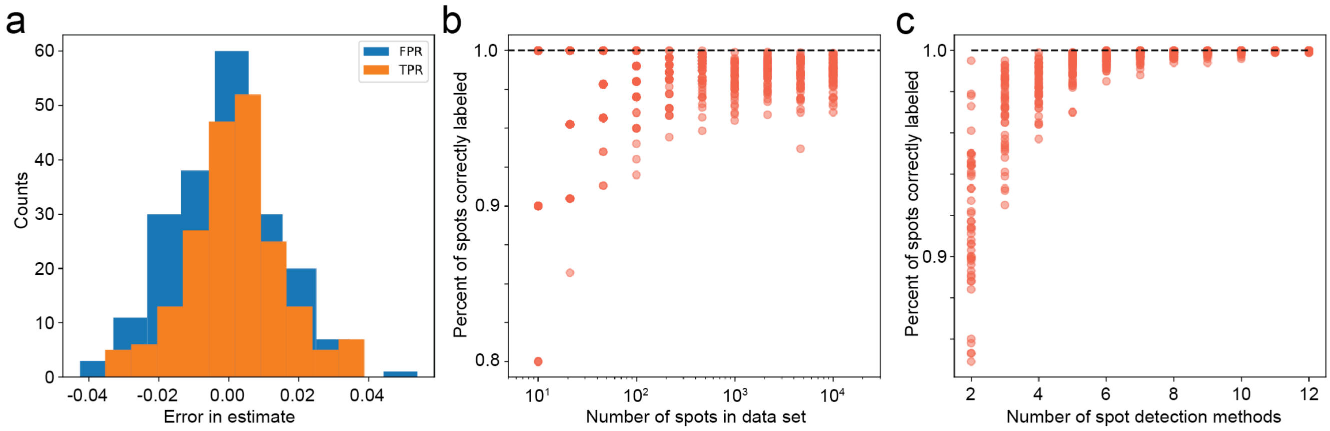

Figure S2: **Benchmarking consensus annotation output of the generative model.** (a) Error distribution for EM estimates of TPR and FPR values for 100 trials with three simulated classical methods. (b) Fraction of simulated detections correctly classified with increasing dataset size (number of spots in the dataset). (c) Fraction of simulated detections correctly classified as a true or false detection by EM for an increasing number of classical spot detection methods used in the EM method.

bioRxiv preprint doi: https://doi.org/10.1101/2023.09.03.556122; this version posted September 8, 2023. The copyright holder for this preprint (which was not certified by peer review) is the author/funder, who has granted bioRxiv a license to display the preprint in perpetuity. It is made available under aCC-BY 4.0 International license.

5.2 Supplementary Note 2: Custom loss function for Polaris' deep learning model for spot detection

We trained the network using a custom loss function composed of a classification loss and a regression loss, which considers the outputs of both of the model's prediction heads. The loss function has the following form:

$$L(y, \hat{y}) = L_{\text{cla}}(C, \hat{C}) + L_{\text{reg}}(R, \hat{R}) \tag{14}$$

where C is the classification head output, R is the regression head output, and $y = (C, R)$. The classification loss is the weighted cross-entropy with inverse class frequency-based weights. The regression loss is given by:

$$L_{\text{reg}}(R, \hat{R}) = \frac{1}{|G_d|} \sum_{i \in G_d} \ell((dy_i, dx_i), (\hat{dy}_i, \hat{dx}_i)) \tag{15}$$

where $R_i = (dy_i, dx_i)$ (i denotes a single pixel), $G_d = \{\text{pixels } i = (i_y, i_x) |$ a spot-containing pixel j exists with $L_\infty(i, j) = \max_{k \in x,y} |i_k - j_k| \leq d\}$, and ℓ is the smooth L_1 function. dx_i is the x-coordinate of the position difference between the nearest spot to pixel i, and pixel i's center. Similarly, dy_i is the y-coordinate of this position difference. d is a configurable parameter which determines the threshold distance from the nearest spot under which the estimated nearest spot's position for that pixel is taken into account in the loss function.

bioRxiv preprint doi: https://doi.org/10.1101/2023.09.03.556122; this version posted September 8, 2023. The copyright holder for this preprint (which was not certified by peer review) is the author/funder, who has granted bioRxiv a license to display the preprint in perpetuity. It is made available under aCC-BY 4.0 International license.

5.3 Supplementary Note 3: Generalization of Polaris' spot detection model to a variety of spot images

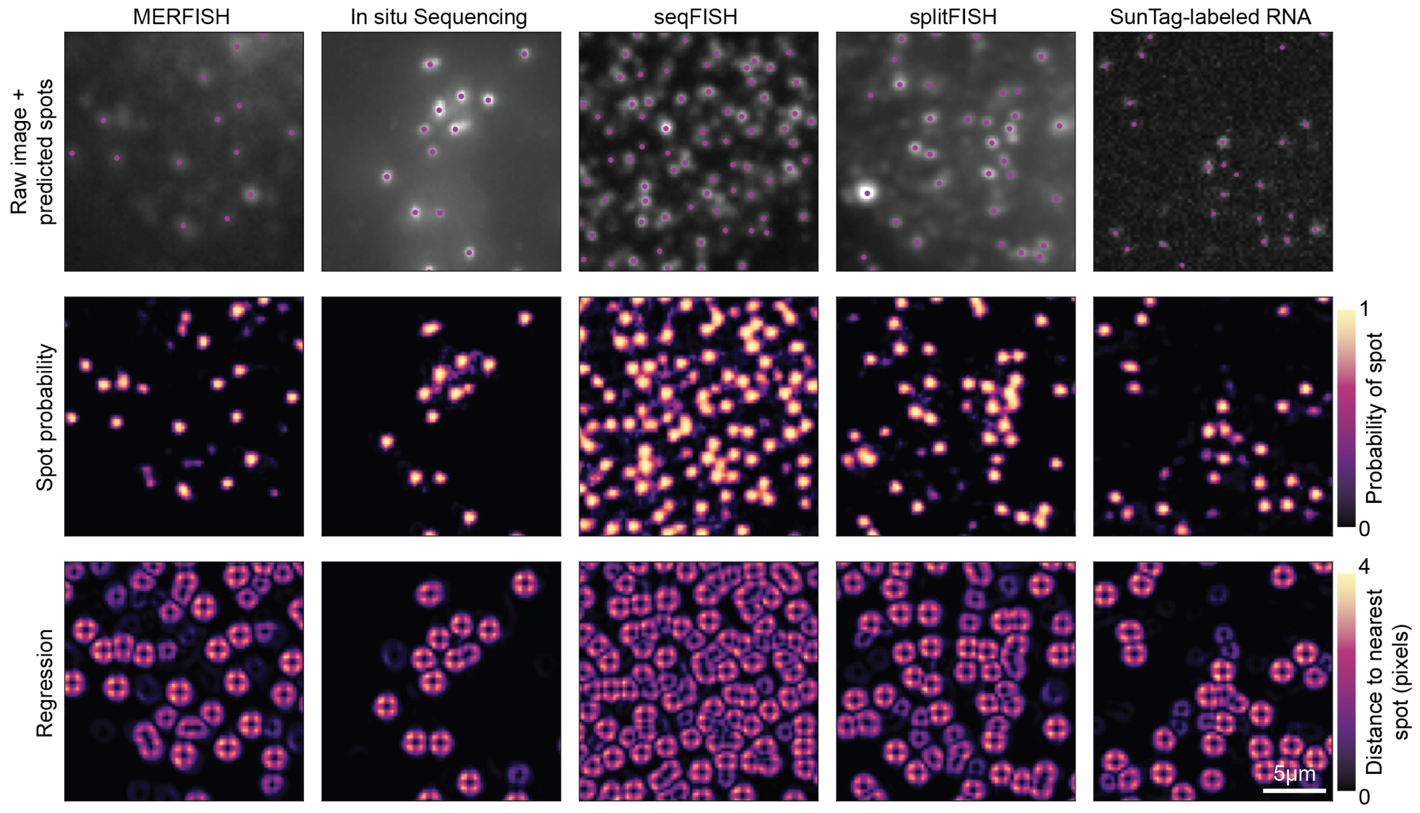

Figure S3: **Polaris' spot detection model generalizes to spot images generated with a variety of single-molecule assays.** The spot probability prediction images encode the pixel-wise spot probability, and the regression prediction images. The regression image is the sum of the square of the subpixel distances to the nearest spot in the x- and y-dimensions. Pixels beyond a threshold value are set to zero. These outputs are used together to generate a set of predicted spot locations with subpixel resolution, plotted over the raw image.

bioRxiv preprint doi: https://doi.org/10.1101/2023.09.03.556122; this version posted September 8, 2023. The copyright holder for this preprint (which was not certified by peer review) is the author/funder, who has granted bioRxiv a license to display the preprint in perpetuity. It is made available under aCC-BY 4.0 International license.

5.4 Supplementary Note 4: Mutual nearest-neighbor method for matching sets of spots

To more quantitatively benchmark the performance of our deep learning models, we need a method for comparing sets of coordinate spot locations. Our method finds sets of mutual nearest neighbors to compare sets of ground-truth and predicted spot locations. Locations that can be matched between ground-truth spots and detected spots are considered true positive detections. Detected spots that cannot be matched to ground-truth spots are false positive detections, and ground-truth spots that cannot be matched to detected spots are false negative detections. These values can be used to calculate metrics such as precision and recall to quantify the performance of a spot detection method.

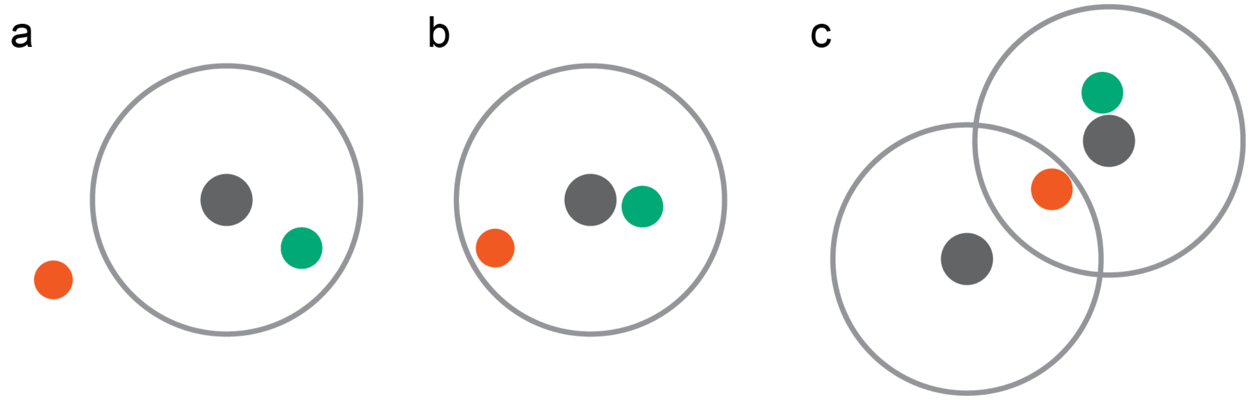

Figure S4: **Example cases handled by a mutual nearest-neighbor matching algorithm.** (a) Example with spots inside and outside the threshold distance to a ground-truth spot. Ground truth spots and their threshold distance are shown in grey. True positive detections are shown in green and false positive detections are shown in orange. (b) Example with two spots inside the threshold distance to a ground-truth spot. (c) Example with two spots within the threshold distance of two ground-truth spots.

To be considered a true detection, a detection must be within some threshold distance of a ground-truth detection. All spots outside this threshold distance are considered false detections (Supplementary Fig. S4a). If more than one detection is within the threshold distance of a ground-truth spot, the detection that is the closest to the ground-truth spot is considered a true detection, and all others are considered false detections (Supplementary Fig. S4b). If more than one detection is within the threshold distance for more than one ground-truth spot, edge cases may arise. For a detection to be considered a true detection, that detection and the corresponding ground-truth spot must be mutual nearest neighbors. Therefore, if a detection is within the threshold distance for two ground-truth spots, it is only paired with a ground-truth spot if they are each others' mutual nearest neighbors. Otherwise, the detection is considered a false detection, even if the detection is within the threshold distance of a ground-truth spot (Supplementary Fig. S4c).

bioRxiv preprint doi: https://doi.org/10.1101/2023.09.03.556122; this version posted September 8, 2023. The copyright holder for this preprint (which was not certified by peer review) is the author/funder, who has granted bioRxiv a license to display the preprint in perpetuity. It is made available under aCC-BY 4.0 International license.

5.5 Supplementary Note 5: Inter-algorithm agreement of spot detection results

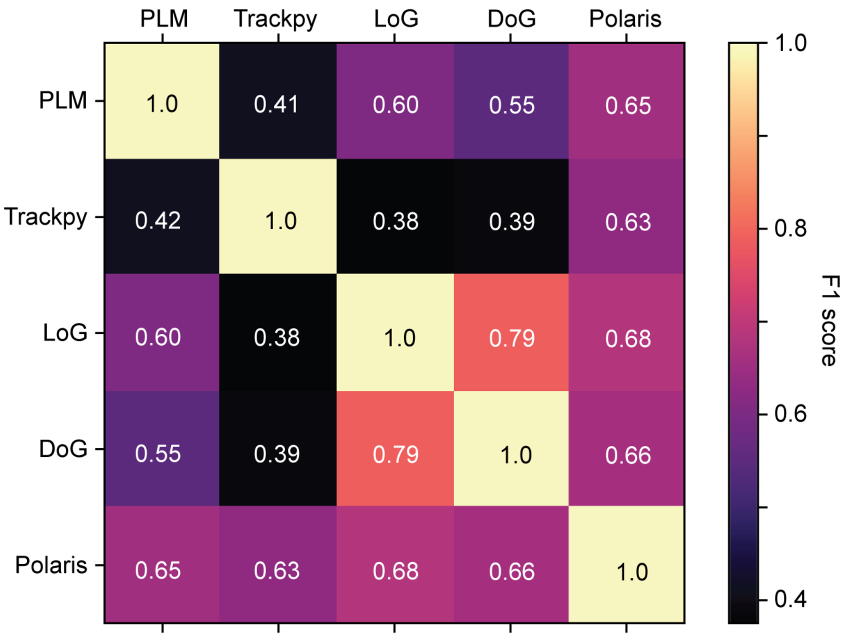

Figure S5: **Quantification of agreement between Polaris' deep learning model and different classical spot detection methods.** The benchmarked methods include maximum-intensity filtering (PLM), the Crocker-Grier centroid-finding algorithm (trackpy), Laplacian of Gaussian (LoG), difference of Gaussians (DoG), and Polaris (DL model).

bioRxiv preprint doi: https://doi.org/10.1101/2023.09.03.556122; this version posted September 8, 2023. The copyright holder for this preprint (which was not certified by peer review) is the author/funder, who has granted bioRxiv a license to display the preprint in perpetuity. It is made available under aCC-BY 4.0 International license.

5.6 Supplementary Note 6: Benchmarking the receptive field of Polaris' spot detection model

To quantify the effect of the receptive field parameter of Polaris' spot detection model on its performance, we followed prior work[27,28] and used simulated spot images, which is a common practice in the field. This approach to benchmarking has the advantage of having an unambiguous ground truth. We find that the receptive field parameter has a modest effect on the model's performance. The model's precision decreases with increasing receptive field, a trend that was also reflected in the model's F1 score across different values for the receptive field (Supplementary Fig. S6a,c). Intermediate values for the receptive field had the lowest recall, but the best values for the validation loss during training (Supplementary Fig. S6b,d). Because the effect of the receptive field parameter on the average value for all metrics is relatively modest, the intermediate value of 13 was selected as the default value for the spot detection model.

bioRxiv preprint doi: https://doi.org/10.1101/2023.09.03.556122; this version posted September 8, 2023. The copyright holder for this preprint (which was not certified by peer review) is the author/funder, who has granted bioRxiv a license to display the preprint in perpetuity. It is made available under aCC-BY 4.0 International license.

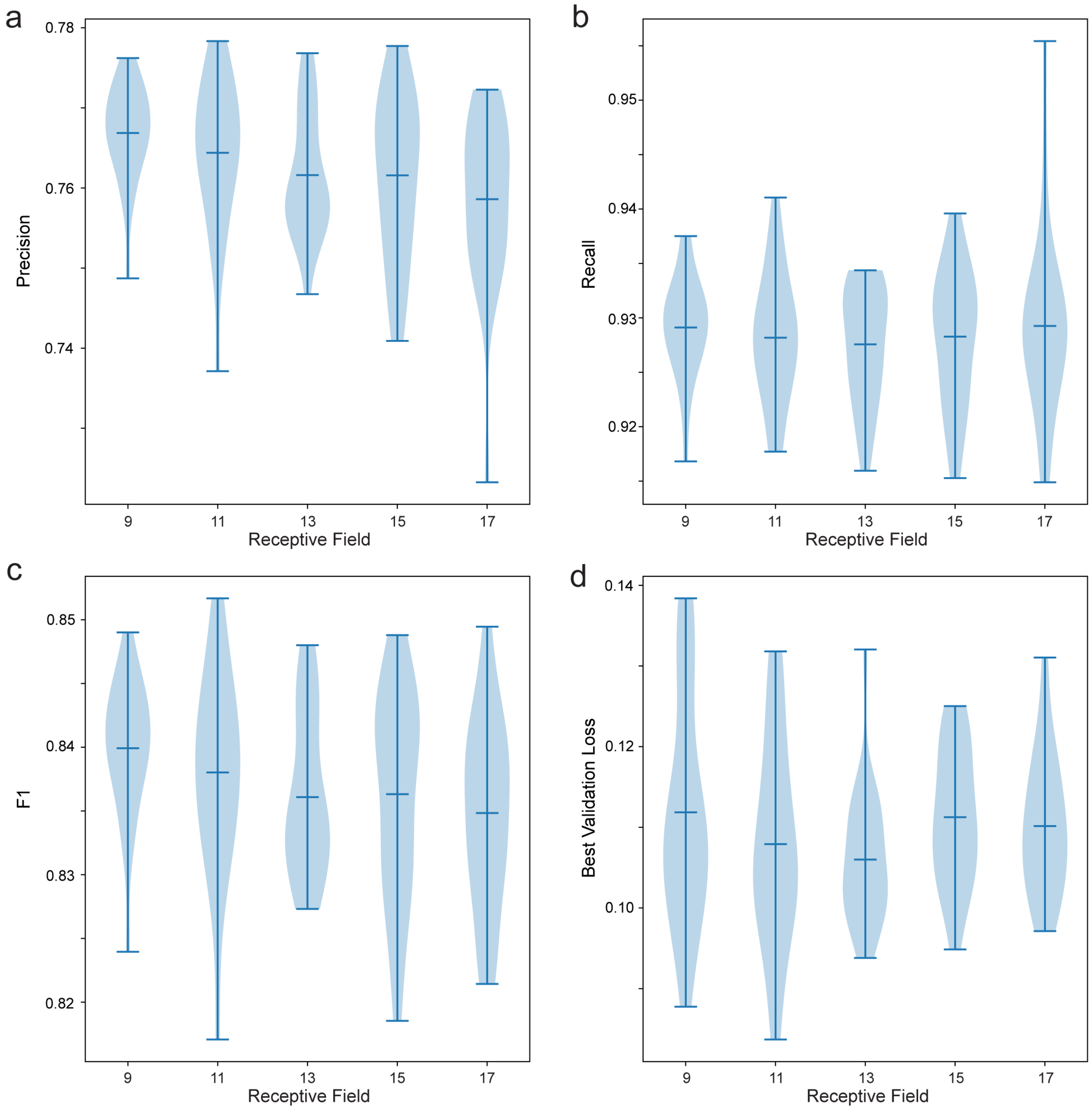

Figure S6: **Benchmarking the receptive field parameter of Polaris' spot detection model.** (a-d) Violin plot quantifying the performance metrics ((a) precision, (b) recall, (c) F1, (d) best validation loss during training) for models trained with different values for receptive field of Polaris' spot detection model. n=24 trained models per receptive field condition.

bioRxiv preprint doi: https://doi.org/10.1101/2023.09.03.556122; this version posted September 8, 2023. The copyright holder for this preprint (which was not certified by peer review) is the author/funder, who has granted bioRxiv a license to display the preprint in perpetuity. It is made available under aCC-BY 4.0 International license.

5.7 Supplementary Note 7: Benchmarking Polaris' spot detection model on simulated images with ranging spot characteristics

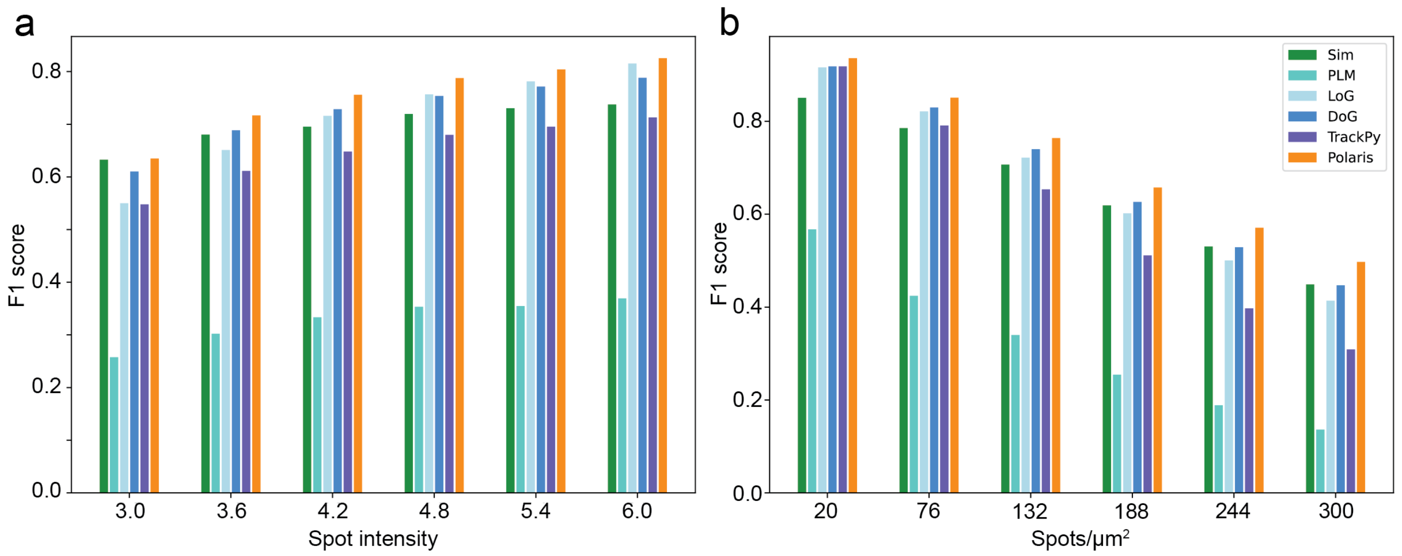

Figure S7: **Benchmarking model performance on simulated spot images with a range of spot intensities and densities.** (a) Performance quantification for models with various training datasets applied to images with varying spot intensity. (b) Performance quantification for models with various training datasets applied to images with varying spot density. Units of spot intensity are arbitrary.

bioRxiv preprint doi: https://doi.org/10.1101/2023.09.03.556122; this version posted September 8, 2023. The copyright holder for this preprint (which was not certified by peer review) is the author/funder, who has granted bioRxiv a license to display the preprint in perpetuity. It is made available under aCC-BY 4.0 International license.

5.8 Supplementary Note 8: Robustness of Polaris' barcode decoding method to dropout

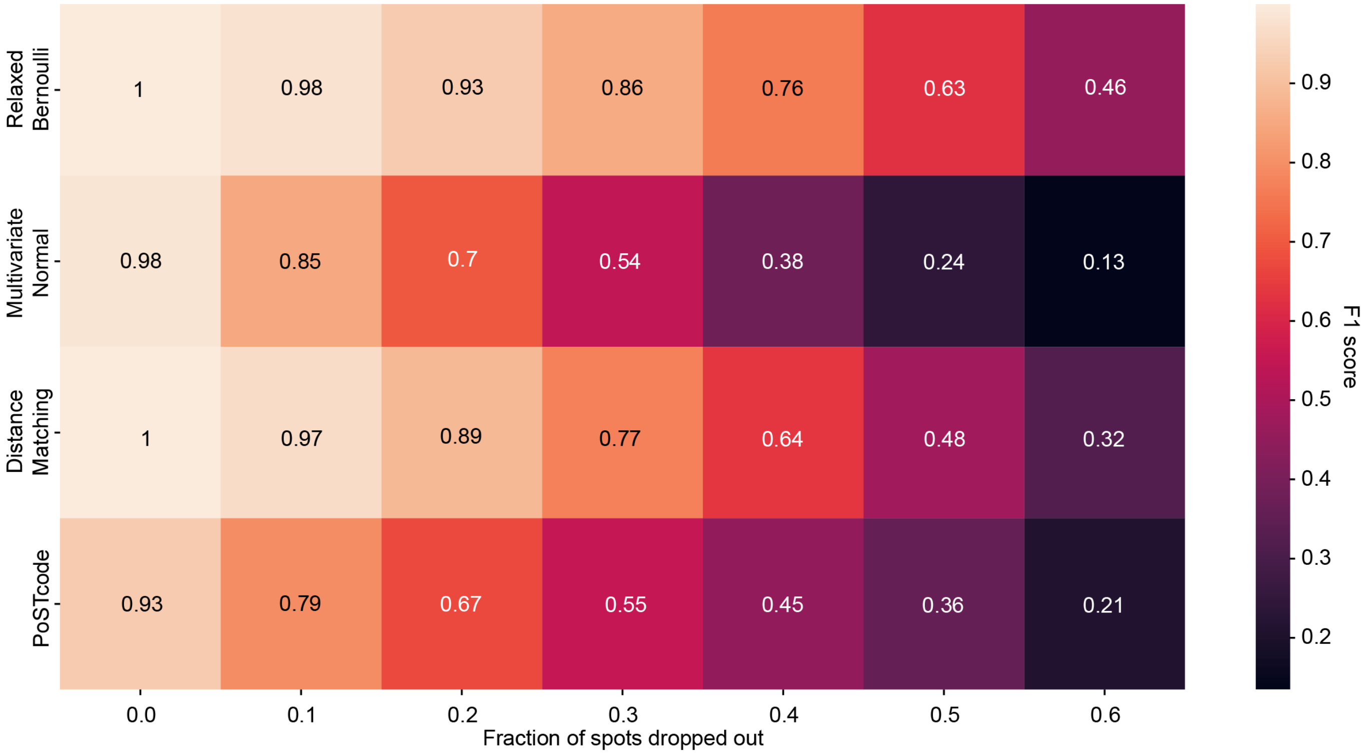

Figure S8: **Benchmarking of robustness of gene decoding methods to dropout.** Quantification of F1 score for four barcode decoding methods (a graphical models of relaxed Bernoulli distributions, a graphical model of multivariate normal distributions, Hamming distance matching, and PoSTcode) for simulated barcode pixel values with a range of dropout rates.

bioRxiv preprint doi: https://doi.org/10.1101/2023.09.03.556122; this version posted September 8, 2023. The copyright holder for this preprint (which was not certified by peer review) is the author/funder, who has granted bioRxiv a license to display the preprint in perpetuity. It is made available under aCC-BY 4.0 International license.

5.9 Supplementary Note 9: Benchmarking Polaris' performance on various multiplexed FISH image sets

We benchmarked Polaris' performance on two previously published MERFISH datasets[29,30] and one seqFISH dataset. We demonstrate that Polaris accurately detects and decodes FISH spots in cell culture and tissue samples (Supplementary Fig. S9a-b). Visual inspection of Polaris' output illustrates the value of multiplexed FISH methods, which quantify gene expression while retaining the sub-cellular location of each transcript. In the kidney tissue sample, anatomical structures create tissue-level organization of gene expression (Supplementary Fig. S9a). These structures would be lost during the tissue homogenization required for sequencing-based assays.

The cultured macrophage sample was created by stimulating immortalized macrophages with lipopolysaccharide (LPS). In response, the macrophages demonstrate cell-to-cell and sub-cellular heterogeneity in inflammatory gene expression (Supplementary Fig. S9b). While single-cell sequencing technologies would be able to capture the cell-to-cell heterogeneity in gene expression, the sub-cellular spatial distribution of transcripts would be lost. Furthermore, image-based spatial transcriptomics assays can be more easily paired with other imaging assays, such as dynamic observation of a live cell reporter or spatial proteomics. These paired measurements allow multiple nodes of an individual cell's signaling state to be observed.

To benchmark Polaris' performance on MERFISH data in tissue samples, we compare its output to counts measured with RNA-seq. We find that for the mouse ileum MERFISH dataset[29], Polaris' mean gene expression counts per cell correlate well with RNA-seq counts (r=0.582), which is similar to the correlation between the counts from the original analysis of this data with RNA-seq counts (r=0.683) (Supplementary Fig. S10a). The counts from these two analyses are also highly correlated (r=0.871) (Supplementary Fig. S10b). For the mouse kidney MERFISH dataset[30], we find similar results; Polaris and the original analysis yield mean gene expression counts per cell that are similarly correlated with RNA-seq counts (r=0.552 and r=0.565, respectively) (Supplementary Fig. S10c). The counts from the two MERFISH analyses are also highly correlated (r=0.954) (Supplementary Fig. S10d). These comparisons demonstrate that Polaris can output a similar result as the original analysis pipelines but without fine-tuning of the underlying spot detection method.

Furthermore, we demonstrate Polaris' performance on seqFISH data in a cell culture sample. We compare its output mean gene expression counts per cell to counts obtained with the Spatial Genomics analysis software and found the two analyses yielded counts that were similarly correlated with RNA-seq counts (r=0.802 and r=0.694, respectively) (Supplementary Fig. S10e). As with the MERFISH analyses, the counts from the two seqFISH analysis pipelines were highly correlated (r=0.909) (Supplementary Fig. S10f). This analysis demonstrates that Polaris generalizes between different FISH assays and sample types without manual parameter tuning. However, the spatial transcriptomics field acknowledges that FISH counts are prone to overdispersion, limiting the utility of comparisons made with a linear regression movdel[30,39].

bioRxiv preprint doi: https://doi.org/10.1101/2023.09.03.556122; this version posted September 8, 2023. The copyright holder for this preprint (which was not certified by peer review) is the author/funder, who has granted bioRxiv a license to display the preprint in perpetuity. It is made available under aCC-BY 4.0 International license.

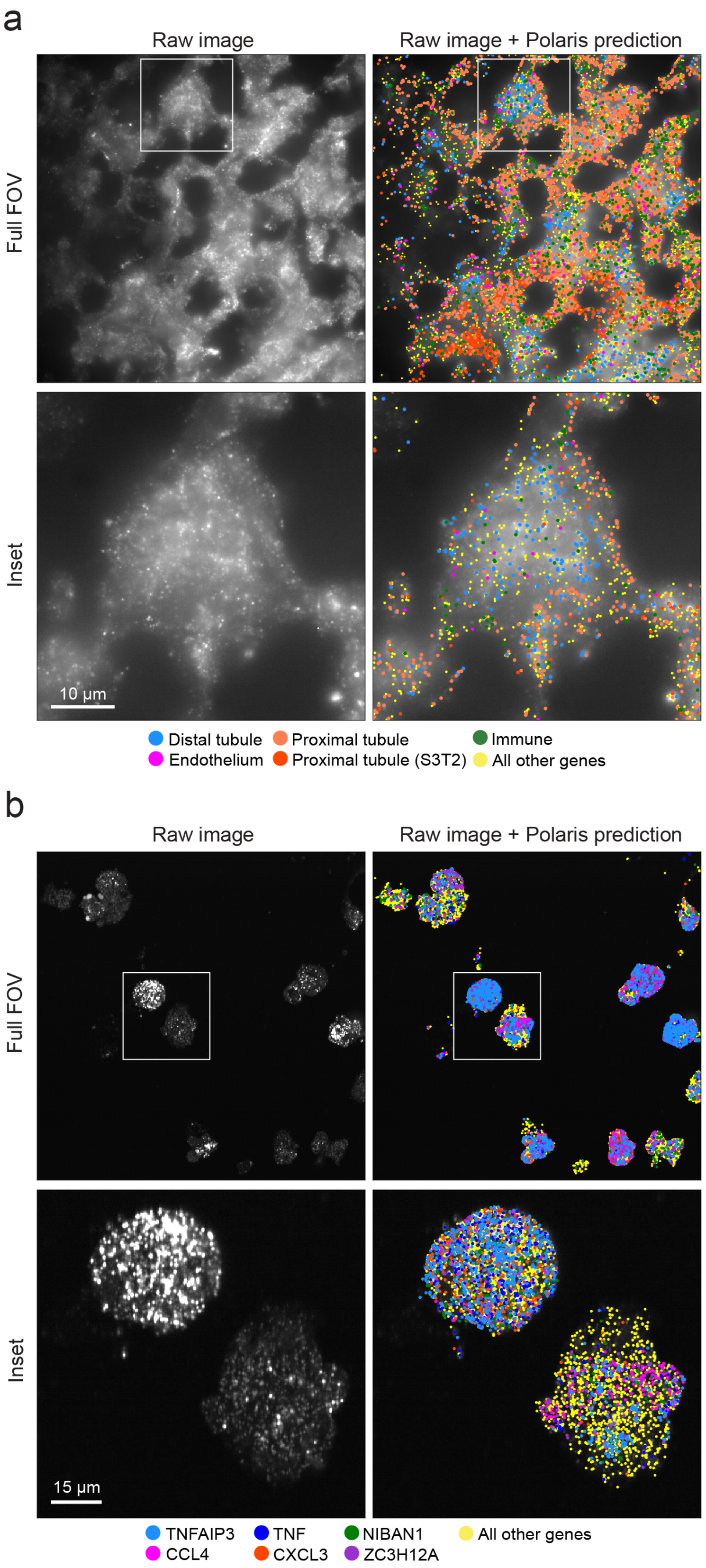

Figure S9: **Demonstration of Polaris' performance on a MERFISH and seqFISH data.** (a) Example Polaris prediction for a MERFISH experiment in a mouse kidney tissue sample.[30] (b) Example Polaris prediction for a seqFISH experiment in a macrophage cell culture sample. The spot colors of the Polaris prediction in (a,b) denote the predicted gene identities. The inset image location is defined by the white box in the full field of view (FOV).

bioRxiv preprint doi: https://doi.org/10.1101/2023.09.03.556122; this version posted September 8, 2023. The copyright holder for this preprint (which was not certified by peer review) is the author/funder, who has granted bioRxiv a license to display the preprint in perpetuity. It is made available under aCC-BY 4.0 International license.

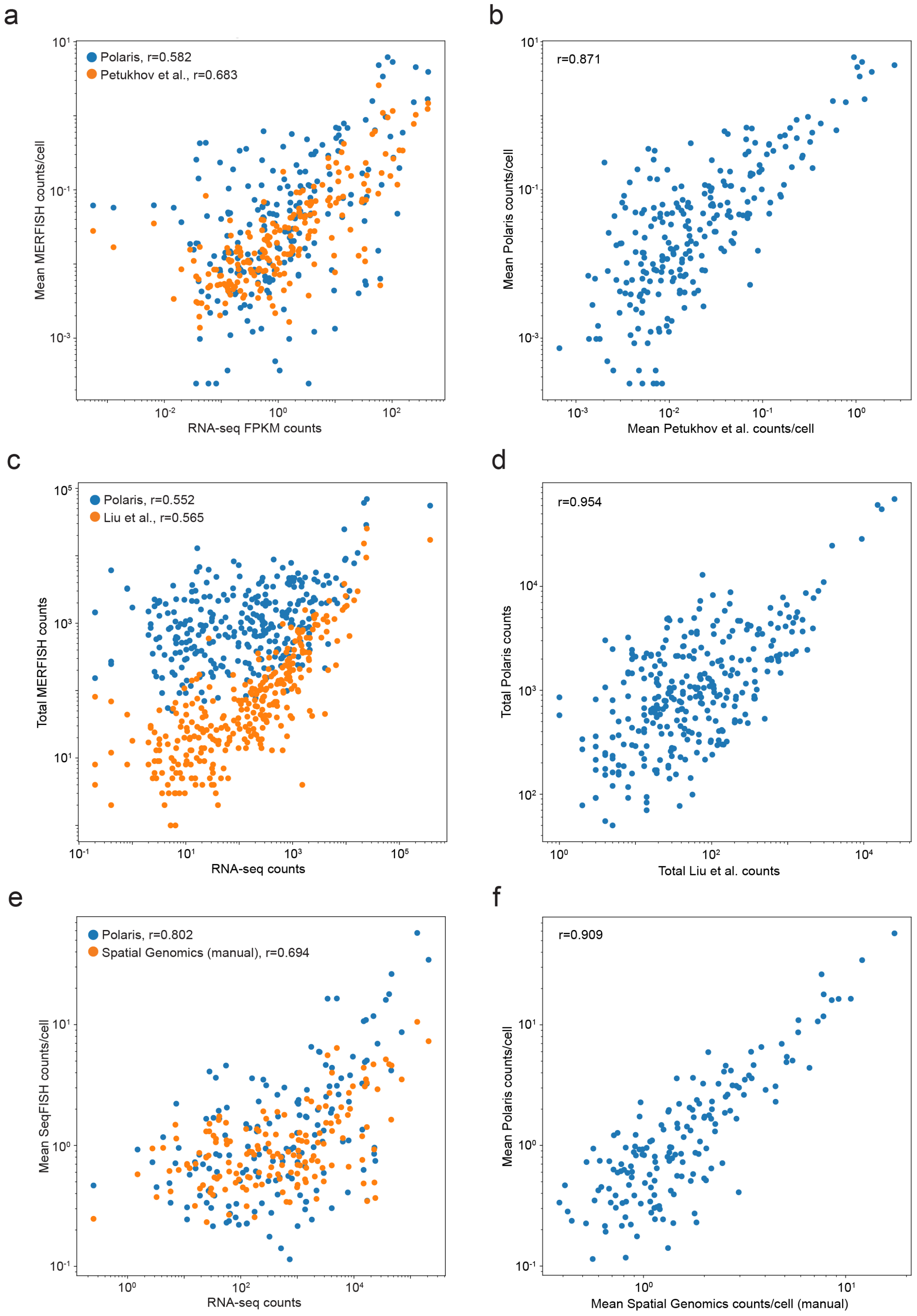

Figure S10: **Correlation of Polaris' quantification of MERFISH data with other quantification methods.** (a,c) Scatter plot plotted in logspace, comparing gene expression counts quantified with MERFISH with counts measured with RNA-seq. (b,d) Scatter plot plotted in logspace, comparing previously published MERFISH gene expression counts with counts quantified with Polaris. (e) Scatter plot plotted in logspace, comparing mean gene expression counts per cell quantified with seqFISH with counts measured with RNA-seq. (f) Scatter plot plotted in logspace, comparing mean gene counts per cell obtained by manual analysis of seqFISH data with gene counts quantified with Polaris.

bioRxiv preprint doi: https://doi.org/10.1101/2023.09.03.556122; this version posted September 8, 2023. The copyright holder for this preprint (which was not certified by peer review) is the author/funder, who has granted bioRxiv a license to display the preprint in perpetuity. It is made available under aCC-BY 4.0 International license.

5.10 Supplementary Note 10: Demonstration of Polaris' performance decoding a ISS barcode library

We benchmarked Polaris' combinatorial ISS barcode assignment performance on a previously published image set [31] in HeLa cells created with pooled optical genetic screen assay, which uses ISS to encode a genetic perturbation (Supplementary Fig. S11a). We find that the barcode counts decoded by Polaris are highly correlated with the counts quantified in the original analysis (r=0.956) (Supplementary Fig. S11b). For all barcodes, Polaris consistently yields higher counts of decoded spots, demonstrating higher sensitivity than the originally published image analysis pipeline.

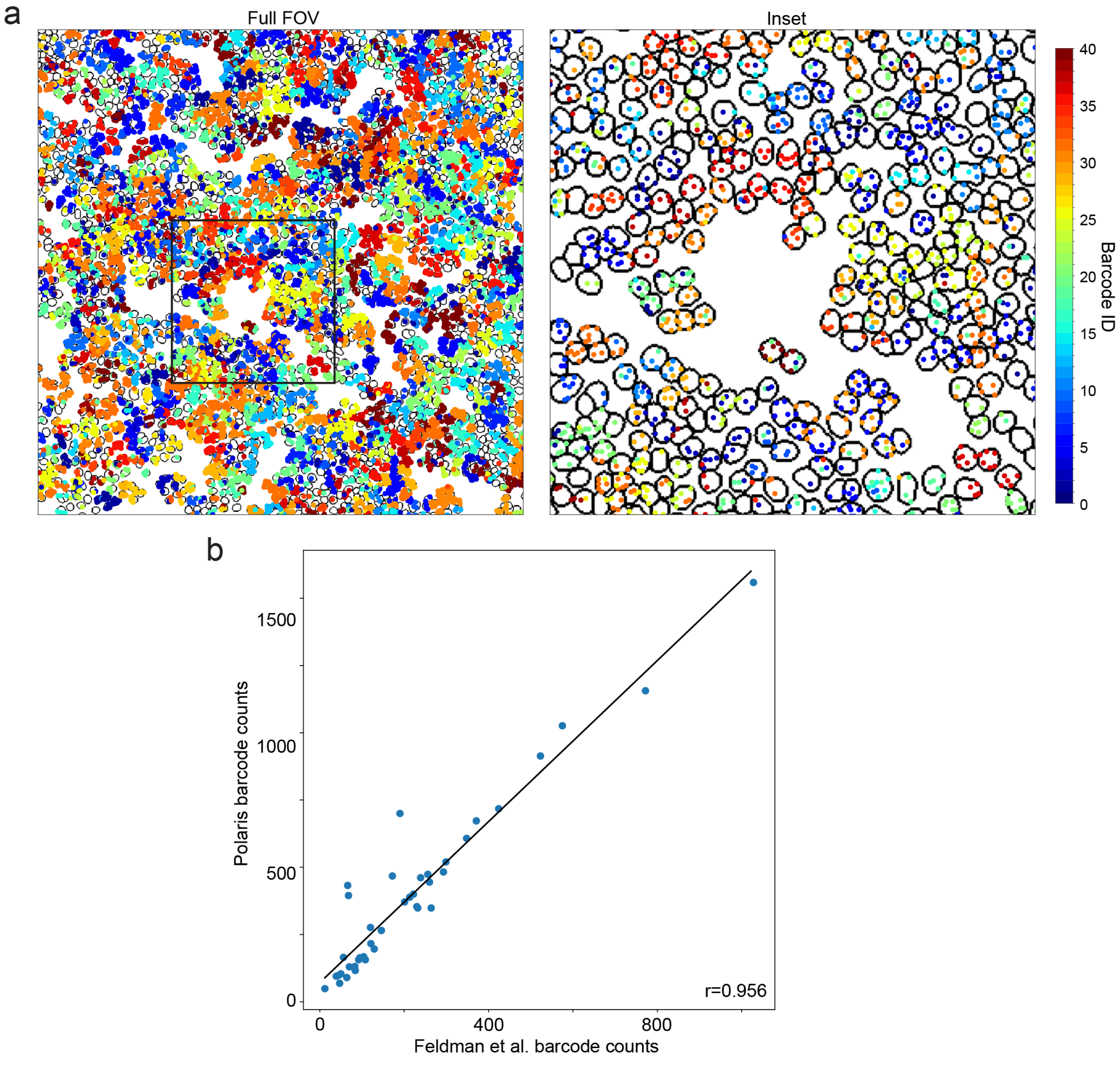

Figure S11: **Demonstration of Polaris' performance on an ISS dataset in HeLa cells.** (a) Example Polaris prediction for the ISS sample. The spot colors correspond with barcode identities. The inset location is defined by the black box in the full field of view (FOV). (b) Scatter plot correlating total counts for each barcode decoded by the original published analysis with counts quantified by Polaris (r=0.956).
